# Socioeconomic Status and Rate of Poverty in Overweight and Obesity among Spanish Children and Adolescents

**DOI:** 10.3390/children11081020

**Published:** 2024-08-21

**Authors:** Alejandra Gallego, Jorge Olivares-Arancibia, Rodrigo Yáñez-Sepúlveda, Héctor Gutiérrez-Espinoza, José Francisco López-Gil

**Affiliations:** 1 Department of Applied Economics, Faculty of Economics and Business, University of Murcia, 30100 Murcia, Spain; alejandra1703200@gmail.com; 2 AFySE Group, Research in Physical Activity and School Health, School of Physical Education, Faculty of Education, Universidad de las Américas, Santiago 7500975, Chile; jolivares@udla.cl; 3 Faculty Education and Social Sciences, Universidad Andres Bello, Viña del Mar 2520000, Chile; rodrigo.yanez.s@unab.cl; 4 One Health Research Group, Universidad de Las Américas, Quito 170124, Ecuador; josefranciscolopezgil@gmail.com; 5 Department of Communication and Education, Universidad Loyola Andalucía, 41704 Seville, Spain

**Keywords:** adiposity, body fat, preschoolers, economic factors, food insecurity

## Abstract

Background: This study aimed to analyze the relationship between socioeconomic status (SES), poverty rate, and the prevalence of overweight/obesity or obesity in children and adolescents aged 2–14. Methods: Parents or guardians reported the weight and height of participants, used to calculate body mass index (BMI) and BMI z-scores according to the International Obesity Task Force standards. Participants were categorized into “overweight/obesity” and “no overweight/obesity” and further into “obesity” and “no obesity”. The rate of poverty rate was determined using data from the National Statistics Institute of Spain, defining it as the percentage of people with income below 60% of the national median. SES was based on the head of household’s occupation and categorized into low, medium, and high levels. Results: Adjusted multilevel models showed participants with medium or high SES had lower odds of overweight/obesity compared to those with low SES (medium SES: odds ratio [OR]: 0.63, 95% confidence interval [CI]: 0.54–0.73; high SES: OR: 0.59, 95% CI: 0.49–0.70). Participants in the high-poverty group had higher odds of having overweight/obesity (OR: 1.40, 95% CI: 1.13–1.74) compared to the low-poverty group. Conclusions: The study highlights significant socioeconomic disparities in childhood overweight/obesity, emphasizing the potential impact of SES and poverty on health outcomes in Spanish children and adolescents.

## 1. Introduction

Childhood and adolescent obesity has been a serious concern for decades due to the chronic and adverse health outcomes associated with it [1,2]. Children and adolescents with obesity are at a significantly higher risk of experiencing unwanted health problems in adulthood, such as cardiovascular diseases, hypertension, and type 2 diabetes [3]. Phelps et al. [2] showed that obesity was more prevalent than thinness among school-aged children and adolescents in the majority of countries, affecting girls in 67% of countries and boys in 63%. The overall increase in the double burden of malnutrition was mainly due to rising obesity rates, while decreases were largely due to reductions in underweight or thinness. Data estimate that obesity prevalence will exceed 20% in most European countries by 2025 [4]. In Spain, data from the *Estudio de Vigilancia de la ALimentación, Actividad física, Desarrollo INfantil y Obesidad en España* (ALADINO) 2019 showed a prevalence of overweight/obesity, overweight, and obesity of 32.9%, 10.9%, and 22.0%, respectively, among Spanish children aged 6 to 9 years [5], based on the International Obesity Task Force cut-off points [6]. Additionally, a longitudinal study by De Bont et al. [7] found that the overall prevalence of childhood obesity in Spain increased from 0.8% at two years of age (in both sexes) to a peak of 17.3% at seven years in girls and 24.1% at nine years in boys. Despite this high prevalence, the World Health Organization (WHO) has estimated that no European country will achieve the proposed goals of reversing rising levels of overweight/obesity by 2025 [8]. Therefore, it is important to efficiently develop specific interventions and policies to curb overweight and obesity in Europe. To develop such interventions, it is necessary to identify the correlates of overweight and obesity. Although there is a multitude of known correlates of overweight and obesity, one potentially important but understudied factor is the poverty rate.

From a socio-ecological perspective, poverty is a significant determinant of child morbidity and mortality globally. Several studies have shown that children from lower-income families have higher rates of obesity and overweight compared to their counterparts from higher-income families [9,10,11]. These disparities can be attributed to factors such as limited access to healthy foods, fewer opportunities for physical activity, and greater exposure to obesogenic environments [12,13]. Lower-income families face significant health challenges due to these limitations, resulting in higher rates of obesity and related health issues [13]. The relationship between poverty and childhood obesity can be explained through a multifactorial model that considers individual, family, and environmental elements [14]. At the individual level, caloric intake and dietary patterns play an important role; children in poverty often consume high-calorie but nutrient-poor diets due to the lower cost of less healthy foods [9]. Additionally, the level of physical activity tends to be lower in these groups due to a lack of safe spaces to play and exercise [10]. A longitudinal study by Lee et al. [15] found that childhood poverty is consistently associated with higher body mass index (BMI) throughout adolescence. At the family level, parents’ education and knowledge about nutrition are significant determinants. Parents with lower educational levels may have less knowledge about the importance of a balanced diet and physical activity, which can influence their children’s food choices and lifestyle [16]. Kakinami et al. [17] highlighted that the latent effects of poverty on childhood adiposity can persist even when socioeconomic conditions improve. At the environmental level, disadvantaged neighborhoods often lack infrastructure that supports healthy lifestyles; for example, the limited availability of supermarkets offering fresh and healthy products, coupled with a high density of fast-food establishments, creates an obesogenic environment that promotes overweight and obesity [18]. Additionally, food insecurity, defined as inconsistent or uncertain access to sufficient and nutritious food, is more prevalent in low-income families and is associated with higher odds of obesity [19]. Banjari et al. [20] identified correlations between poverty and obesity in seven-year-old children in Croatia and Montenegro, highlighting the potential influence of socioeconomic factors on weight.

Public policies play a significant role in mitigating childhood obesity, especially among socioeconomically disadvantaged groups. Programs that improve access to healthy foods and promote physical activity can have a significant impact [21]. For example, initiatives that subsidize fruits and vegetables for low-income families, as well as school programs offering healthy meals and exercise opportunities, have been effective in reducing the prevalence of childhood obesity [22]. In Spain, the *Estrategia para la Nutrición, ctividad Física y Prevención de la Obesidad* (NAOS) has been a comprehensive effort to address childhood obesity through multiple interventions. However, despite these efforts, childhood obesity remains a relevant issue, suggesting the need for more specific approaches targeting the most vulnerable groups. Hernandez and Pressler [23] emphasized that the accumulation of poverty during childhood has lasting effects on overweight and obesity in young adulthood, indicating the need for early and sustained interventions.

Based on the above, this study aimed to analyze the relationship between individual economic factors (i.e., socioeconomic status [SES]) and contextual economic factors (i.e., poverty rate) and the prevalence of overweight and obesity in children and adolescents. This analysis is important to identify the populations at highest risk and to develop more effective and equitable interventions. By focusing on children, this study also seeks to contribute to the understanding of how socioeconomic conditions in early life stages can influence long-term health.

## 2. Materials and Methods

### 2.1. Population and Study Design

This comprehensive cross-sectional study utilized data from the Spanish National Health Survey 2017, managed by the Ministry of Health, Consumer Affairs, and Social Welfare in collaboration with the National Statistics Institute [24,25]. The survey employed a three-tiered sampling method: initially selecting census sections, then households, and finally individuals. From each household, one adult (aged 15 or older) was chosen to complete the Adult Questionnaire, and if the household included children aged 0–14, one child was randomly selected to complete the Minor Questionnaire by the parents or guardians of children. Participants were informed about the survey through a letter from the Ministry, detailing the objectives, the voluntary and anonymous nature of the study, and notifying them of an impending visit from a certified interviewer.

This research focused specifically on data from the Minor Questionnaire, targeting children aged 0–14 years. The initial sample included 6101 participants (100%). However, 1273 participants (20.8%) were removed due to incomplete, missing information on anthropometric data. Furthermore, 371 (6.1%) participants were eliminated because of a lack of information on various covariates (e.g., SES, diet quality). The final sample included 4462 Spanish children and adolescents aged 2–14 years (73.1%).

The data for this study were provided by the Ministry of Health, Consumer Affairs, and Social Welfare and are publicly accessible on the official Spanish Government website [24]. Ethical committee approval was not required according to Spanish law, as the data used were secondary and anonymized.

### 2.2. Procedures

#### 2.2.1. Measurement of Overweight/Obesity (Dependent Variable)

The weight and height of the participants were reported by parents or guardians. These data were used to calculate the BMI, which was then converted into BMI z-scores according to the standards of the International Obesity Task Force (IOTF) adjusted for sex and age [6]. Minors were classified into four categories based on their BMI z-score: (a) thinness, (b) normal weight, (c) overweight, and (d) obesity. For this study, we focused on the analysis of overweight and obesity combined, categorizing participants into two groups: “overweight/obesity” and “no overweight/obesity” (including thinness and normal weight). Furthermore, another classification was as follows: “obesity” and “no obesity” (including overweight, thinness and normal weight). These classifications are justified due to the importance of addressing the growing prevalence of these conditions and their implications for long-term health [26].

#### 2.2.2. Assessment of the Poverty Rate (Independent Variable)

The poverty rate was calculated using data provided by the National Statistics Institute of Spain. This indicator is defined as the percentage of people whose income is below 60% of the national median equivalent income per consumption unit. Equivalent income is obtained by adjusting the household’s total income according to its size and composition, using the modified Organization for Economic Co-operation and Development (OECD) equivalence scale, which assigns a weight of 1 to the first adult, 0.5 to each additional person aged 14 or older, and 0.3 to each child under 14 years [25]. This methodology allows for the comparison of income levels adjusted to household needs, providing a standardized measure of relative poverty.

#### 2.2.3. Socioeconomic Status (Independent Variable)

The SES was determined by the occupation of the head of the household, encompassing various categories: employed individuals (including salaried employees and self-employed professionals), unemployed persons actively seeking employment, retirees, and pre-retirees reliant on pensions or retirement income, students, individuals permanently incapacitated for work, and those primarily engaged in unpaid household duties. SES was categorized into three levels: low SES (categories 5 and 6), medium SES (categories 3 and 4), and high SES (categories 1 and 2). This classification system facilitates an in-depth examination of how SES impacts the relationship between poverty and obesity [23].

#### 2.2.4. Covariates

Information regarding the child’s age, sex, and immigrant status (whether native-born or foreign-born) was provided by parents or guardians. The child’s SES was evaluated based on the primary adult’s occupation in the household. The choice of the head of household’s occupation as an indicator of the SES is based on its ability to reflect income, education, and social status. Previous research highlights that occupation captures multiple dimensions of the SES, influencing health through access to resources and healthy lifestyles [27,28]. The determination of the head of the household as the main economic provider is common in socioeconomic studies, accurately reflecting the distribution of economic power in the household [29]. To assess physical activity levels, a concise questionnaire adapted from the International Physical Activity Questionnaire was used, focusing on a single question about the child’s leisure time physical activities [30]. Response options included: “no exercise” (engaging in sedentary activities such as reading, watching TV, going to the cinema, etc.); “occasional physical activity or sport”; “physical activity several times a month”; and “sports or physical training several times a week” [30]. Recreational screen time was separately reported for weekdays and weekends using the question: “How much time does your child typically spend on a weekday in front of a screen, including computers, tablets, TVs, videos, video games, or cell phones?” The possible responses were: “no time or almost no time”, “less than one hour”, and “one hour or more”. Recreational screen time recommendations were established based on the WHO international guidelines for children under 5 years (under 2 years: no screen time; 2 to 4 years: ≤1 h/day) and the Canadian guidelines on screen time for young people (5 to 14 years: ≤2 h/day of ST). Additionally, sleep duration was measured by asking the following: “Approximately how many hours does your child usually sleep daily?”. The proportion of young people meeting the sleep recommendations was established following the National Sleep Foundation’s sleep duration guidelines: preschoolers (from 10 to 13 h/day of sleep); children (from 9 to 11 h/day of sleep); adolescents (from 8 to 10 h/day of sleep).

#### 2.2.5. Assessment of Healthy Eating Index (HEI)

Dietary intake data were collected using a validated food frequency questionnaire (FFQ) from the Spanish National Health Survey 2017, administered to the parents or guardians of the participants. The FFQ captured detailed information on the frequency and portion sizes of various food items consumed over the past month. The data obtained from the FFQ were then used to calculate the Healthy Eating Index (HEI) scores, a measure of diet quality that assesses how well an individual’s dietary intake aligns with dietary guidelines [31]. The HEI scores were calculated based on the following components: total fruit, whole fruit, total vegetables, greens and beans, whole grains, dairy, total protein foods, seafood and plant proteins, fatty acids, refined grains, sodium, and added sugars [32]. Each component is scored on a scale from 0 to 10, with higher scores indicating better adherence to dietary guidelines. The total HEI score is the sum of the component scores, with a maximum possible score of 100 [33]. For this study, the HEI scores were categorized into three levels: low (≤50), medium (51–80), and high (>80).

### 2.3. Statistical Analysis

Descriptive data were presented as absolute and relative frequencies for categorical variables or mean (*M*) and SD for continuous variables. Following a previously described methodology [28], generalized linear models with binomial distribution were constructed to analyze the associations of the independent variables on overweight/obesity or obesity in children and adolescents. Therefore, odds ratios (ORs) and their 95% confidence intervals (CIs) were obtained. To assess general contextual effects, we estimated the intraclass correlation coefficient (ICC), which represents the proportion of the total individual variance explained by the country of residence and the median odds ratio (MOR), conceptualized as the higher odds that a participant would have, on average, if they were to move from one area with lower odds to another area with higher odds [29]. In addition, the Proportion of Opposite Odds Ratios (POOR) was also calculated, which indicates the proportion of ORs in the opposite direction to the overall OR [28]. POOR values range from 0 to 50%. A POOR value of 0% means that all ORs have the same sign. A POOR value of 50% means that half of the ORs are of opposite sign, so the association is very heterogeneous. Furthermore, to measure the goodness of fit of our models, we used the Bayesian information criterion (BIC) [30]. Moreover, Nakagawa’s conditional coefficient of determination (*R*^2^) were calculated to obtain the variance explained, including both fixed and random effects [31]. Statistical significance was set at a *p*-value of 0.05. All analyses accounted for survey weights and were conducted using the software R (Version 4.3.0) (R Core Team, Vienna, Austria) and RStudio (Version 2023.03.1) (Posit, Boston, MA, USA).

## 3. Results

The descriptive analysis of the study population revealed significant variations in the prevalence of overweight/obesity according to SES (Table 1). The median age of participants was consistently 9.0 years across all SES groups. Sex distribution was balanced, with boys comprising approximately 51% and girls 49% of the sample across SES status. Immigrant status showed notable differences, with a higher proportion of foreign-born children in the low SES group (7.4%) compared to the medium (3.7%) and high SES groups (1.8%). The median BMI was highest in the low SES group (18.4) compared to the medium (17.6) and high SES groups (17.4). The prevalence of overweight/obesity was significantly higher in the low SES group (37.2%) compared to the medium (26.8%) and high SES groups (25.3%), and obesity prevalence followed a similar trend, with 15.6% in the low SES group, 9.5% in the medium SES group, and 6.8% in the high SES group.

Analysis based on poverty status (Table 2) also showed significant variations in the prevalence of overweight/obesity. SES was more skewed in the high-poverty group, with 50.4% of children in this category compared to 39.3% in the low-poverty group. The mean BMI was highest in the high-poverty group (18.9) compared to the medium (18.4) and low-poverty groups (18.4). The prevalence of overweight/obesity was highest in the high-poverty group (35.8%), followed by the medium (28.9%) and low-poverty groups (28.1%). Obesity prevalence was also highest in the high-poverty group (13.9%) compared to the medium (10.1%) and low-poverty groups (10.5%).

The results of the generalized linear mixed model examining the association of SES and poverty status with overweight/obesity among Spanish children and adolescents are found in Table 3. Young people with low SES had a higher OR for overweight/obesity compared to those from medium and high SES backgrounds. When examining the multilevel model adjusted for covariates, participants with medium SES or high SES had lower odds of overweight/obesity compared to those with low SES (medium SES: OR: 0.63, 95% CI: 0.54–0.73, *p* < 0.001; high SES: OR: 0.59, 95% CI: 0.49–0.70, *p* < 0.001). Additionally, participants in the high-poverty group had higher odds of having overweight/obesity (OR: 1.48, 95% CI 1.17–1.86, *p* = 0.00)) compared to those in the low-poverty group. Additionally, this analysis revealed that immigrant status was also related to overweight/obesity, with foreign-born children showing higher odds (OR: 1.12, 95% CI: 0.83–1.50), although this was not statistically significant (*p* = 0.462). Age had an inverse relationship with overweight/obesity, where each additional year reduced the odds (OR: 0.96, 95% CI: 0.94–0.98, *p* < 0.001). Physical activity levels were related to overweight/obesity, with those engaging in sports or physical training several times a week having higher odds (OR: 1.04, 95% CI: 0.85–1.28), though this was not statistically significant (*p* = 0.721). Meeting with the screen time guidelines was associated with lower odds of overweight/obesity (OR: 0.98, 95% CI: 0.86–1.12) but was not statistically significant (*p* = 0.803).

Table 4 displays the results of the generalized linear mixed model examining the association of SES and poverty status with obesity among Spanish children and adolescents. Children with low SES had higher odds of obesity. In the adjusted multilevel model, medium SES was associated with lower odds of obesity (OR: 0.56, 95% CI: 0.45–0.70, *p* < 0.001), and high SES was linked to even lower odds (OR: 0.37, 95% CI: 0.28–0.50, *p* < 0.001). Children in the high-poverty group had significantly higher odds of obesity (OR: 1.48, 95% CI: 1.18–1.84, *p* = 0.001) in the univariable model, representing the same trend in the multilevel model (OR: 1.40, 95% CI: 1.02–1.94, *p* = 0.039). Immigrant status is also related to obesity, with foreign-born children showing higher odds (OR: 1.29, 95% CI: 0.88–1.91), though this was not statistically significant (*p* = 0.194). Age was inversely associated with obesity, where each additional year reduced the odds (OR: 0.85, 95% CI: 0.82–0.87, *p* < 0.001). Higher levels of physical activity were associated with lower odds of obesity, particularly in those engaging in sports or physical training several times a week (OR: 0.95, 95% CI: 0.71–1.28), though this was not statistically significant (*p* = 0.749). Adherence to sleep guidelines was inversely associated with obesity (OR: 0.86, 95% CI: 0.70–1.06), though this was not statistically significant (*p* = 0.168).

## 4. Discussion

This study provides a detailed assessment of the relationship between SES and the poverty rate status and the prevalence of overweight/obesity or obesity in Spanish minors. These findings are consistent with global studies documenting an increase in childhood obesity, especially in socioeconomically disadvantaged contexts [1]. Regarding socioeconomic factors, our findings indicate that the likelihood of having overweight/obesity or obesity is higher in children from low-income families. This result aligns with scientific literature showing that children from lower-income families have higher rates of obesity [9,12]. The association between SES and the prevalence of childhood obesity can be due to various interrelated factors, such as limited access to healthy foods and reduced opportunities for physical activity [34]. Families with higher incomes typically have more economic resources to purchase nutritious and quality foods [19]. These households can access a greater variety of fresh fruits, vegetables, and lean proteins, contributing to lower energy intake and, thus, a lower likelihood of having overweight/obesity [9]. Similarly, low-income families face economic constraints that limit their access to healthy food options [10]. Additionally, the environment in which children live can influence their food choices and physical activity levels. Areas with higher SES tend to better promote health by attracting certain services (e.g., grocery stores and exercise facilities) and offering physical features conducive to physical activity (e.g., parks and well-maintained streets). In contrast, low-income neighborhoods are associated with a greater availability of fast-food establishments and a lower availability of fruit and vegetable stores, along with limited sports facilities [16].

Another possible reason explaining our results could be related to education and knowledge about nutrition and health. It has been suggested that families with low SES tend to have lower levels of education and knowledge about healthy eating and the importance of maintaining a healthy lifestyle [30]. This lack of knowledge may lead to unhealthier decisions regarding diet and physical activity, which can favor the development of obesity. Families with lower SES might not have the same access to information, educational programs, and health services that promote healthy habits and prevent overweight/obesity [23]. Furthermore, limited awareness about the benefits of nutritious food and regular physical activity could result in poorer dietary choices and a more sedentary lifestyle. Research indicates that individuals from lower socioeconomic backgrounds have less knowledge and less favorable attitudes toward healthy eating and physical activity, which can lead to unhealthy dietary choices and sedentary behavior [35]. Consequently, these factors contribute to higher rates of obesity among children from low-income families [36]. This relationship underscores the importance of improving health education and access to resources that support healthy living, especially in socioeconomically disadvantaged communities. Additionally, improving nutritional education in both schools and homes is critical. Initiatives involving parents and caregivers in nutritional education programs can help mitigate some of the negative effects of poverty on diet and child health [16]. Interventions should be culturally sensitive and tailored to the specific needs of local communities, ensuring they are accessible and relevant to target populations [10].

Another possible justification for these findings is that children and adolescents with low SES and high poverty status are more exposed to obesogenic environments [37]. Previous studies have shown that disadvantaged neighborhoods often lack the infrastructure that supports healthy lifestyles, increasing the likelihood of obesity [4,18,37]. Food insecurity, defined as inconsistent or uncertain access to sufficient and nutritious food, is more prevalent in low-income families and is associated with higher odds of obesity [19]. Other authors identified associations between poverty and obesity in seven-year-old children in Croatia and Montenegro, highlighting the influence of socioeconomic factors on weight [20]. Public policies play a crucial role in mitigating childhood obesity, especially among socioeconomically disadvantaged groups, as programs that improve access to healthy foods and promote physical activity can have a significant impact [21]. For example, initiatives that subsidize fruits and vegetables for low-income families, as well as school programs offering healthy meals and exercise opportunities, have been effective in reducing the prevalence of childhood obesity [22]. In Spain, the *Estrategia* NAOS has been a comprehensive effort to address childhood obesity through multiple interventions. However, the persistence of childhood obesity suggests that more specific approaches targeting the most vulnerable groups are needed. Other studies have emphasized that the accumulation of poverty during childhood has lasting effects on overweight and obesity in young adulthood, indicating the need for early and sustained interventions [23]. To effectively address childhood obesity in poverty contexts, policies should focus on reducing economic barriers to accessing healthy foods and physical activities. Programs that subsidize fruits and vegetables for low-income families have proven effective and should be expanded [21,22].

Despite the limitations of this study, including reliance on self-reported data and its cross-sectional design, our findings underscore the critical importance of addressing socioeconomic and environmental factors in the fight against childhood obesity. The cross-sectional nature prevents establishing causal relationships, highlighting the need for future longitudinal studies to determine how poverty influences obesity over time and assess intervention effectiveness [15]. Furthermore, while previously validated, the brevity of our measures may lack the depth needed for comprehensive data, suggesting that more detailed metrics could yield further insights. Reliance on parent-reported height and weight introduces potential measurement errors and biases, and lifestyle information may suffer from recall or social desirability biases. Despite controlling for key confounders like sex, age, and lifestyle factors, residual confounding may persist. Moreover, this study did not consider the status of adipose tissue rebounding and the stages of puberty, which are significant factors influencing excess weight and obesity in children and adolescents. Future research should incorporate these aspects to provide a more comprehensive understanding of obesity determinants among youth. Nonetheless, the study’s large and representative sample strengthens the external validity of our findings, providing robust evidence of the interplay between individual and contextual economic factors and childhood obesity, thus underscoring the necessity for targeted public health interventions.

## 5. Conclusions

Our study provides a comprehensive analysis of the association between SES, poverty status, and the prevalence of overweight/obesity or obesity among children in Spain, using data from the Spanish National Health Survey 2017. The findings highlight significant socioeconomic disparities in childhood overweight/obesity, underscoring the influence of SES and poverty on health outcomes. Minors with lower SES exhibited a markedly higher prevalence of overweight and obesity compared to their higher SES counterparts, which could be related to certain factors such as limited access to healthy foods and fewer opportunities for physical activity. Poverty status could exacerbate these disparities, with children in high-poverty situations exhibiting the highest rates of overweight and obesity. These results underscore the urgent need for public health interventions aimed at improving access to healthy foods and promoting physical activity, especially for low SES and regions with high poverty rates.

## Figures and Tables

**Table 1:** Descriptive data of the study participants according to socioeconomic status (*N* = 4263).

Variables		Low SES	Medium SES	High SES	*p*-Value
Age	Mean (SD)	8.8 (3.7)	8.5 (3.7)	8.3 (3.9)	0.002
Sex	Boys (%)	981 (50.6)	786 (51.8)	520 (51.5)	0.757
	Girls (%)	957 (49.4)	730 (48.2)	489 (48.5)	
Immigrant status	Native-born (%)	1793 (92.5)	1459 (96.2)	991 (98.2)	<0.001
	Foreign-born (%)	145 (7.5)	57 (3.8)	18 (1.8)	
BMI	Mean (SD)	19.2 (5.1)	18.2 (4.3)	17.8 (3.7)	<0.001
Overweight/obesity	No (%)	1224 (63.2)	1115 (73.5)	755 (74.8)	<0.001
	Yes (%)	714 (36.8)	401 (26.5)	254 (25.2)	
Obesity	No (%)	1642 (84.7)	1375 (90.7)	942 (93.4)	<0.001
	Obesity (%)	296 (15.3)	141 (9.3)	67 (6.6)	
HEI (score)	Mean (SD)	69.1 (9.4)	70.3 (8.9)	72.0 (8.3)	<0.001
PA category	No exercise (%)	424 (21.9)	230 (15.2)	136 (13.5)	<0.001
	Occasional physical activity or sport (%)	508 (26.2)	378 (24.9)	210 (20.8)	
	Physical activity several times a month (%)	519 (26.8)	430 (28.4)	300 (29.7)	
	Sports or physical training several times a week (%)	487 (25.1)	478 (31.5)	363 (36.0)	
ST duration (min)	Mean (SD)	122.5 (75.3)	112.5 (69.1)	102.6 (63.2)	<0.001
ST guidelines	Non-meeting (%)	1101 (56.8)	853 (56.3)	539 (53.4)	0.197
	Meeting (%)	837 (43.2)	663 (43.7)	470 (46.6)	
Sleep duration (hour)	Mean (SD)	9.4 (1.2)	9.5 (1.2)	9.5 (1.2)	0.006
Sleep guidelines	Non-meeting (%)	665 (34.3)	516 (34.0)	336 (33.3)	0.858
	Meeting (%)	1273 (65.7)	1000 (66.0)	673 (66.7)	
Poverty	Mean (SD)	22.4 (8.4)	21.0 (8.0)	20.3 (8.0)	<0.001
Poverty status	Low poverty (%)	616 (31.8)	558 (36.8)	384 (38.1)	<0.001
	Medium poverty (%)	569 (29.4)	498 (32.8)	345 (34.2)	
	High poverty (%)	753 (38.9)	460 (30.3)	280 (27.8)	

BMI, body mass index; HEI, Healthy Eating Index; PA, physical activity; SD, standard deviation; SES, socioeconomic status; ST, screen time.

**Table 2:** Descriptive data of the study participants according to poverty status (N = 4263).

Variables		Low Poverty	Medium Poverty	High Poverty	*p*-Values
Age	Mean (SD)	9.0 (6.0)	9.0 (7.0)	9.0 (7.0)	0.063
Sex	Boys (%)	826 (53.0)	714 (50.6)	747 (50.0)	0.213
	Girls (%)	732 (47.0)	698 (49.4)	746 (50.0)	
SES status	Low SES (%)	616 (39.5)	569 (40.3)	753 (50.4)	<0.001
	Medium SES (%)	558 (35.8)	498 (35.3)	460 (30.8)	
	High SES (%)	384 (24.6)	345 (24.4)	280 (18.8)	
Immigrant status	Native-born (%)	1472 (94.5)	1339 (94.8)	1432 (95.9)	0.165
	Foreign-born (%)	86 (5.5)	73 (5.2)	61 (4.1)	
BMI	Mean (SD)	18.4 (4.3)	18.4 (5.0)	18.9 (4.4)	0.003
Overweight/obesity	No (%)	1132 (72.7)	1004 (71.1)	958 (64.2)	<0.001
	Yes (%)	426 (27.3)	408 (28.9)	535 (35.8)	
Obesity	No (%)	1404 (90.1)	1270 (89.9)	1285 (86.1)	<0.001
	Yes (%)	154 (9.9)	142 (10.1)	208 (13.9)	
HEI (score)	Mean (SD)	69.6 (8.2)	71.6 (8.8)	69.3 (10.0)	<0.001
PA category	No exercise (%)	187 (12.0)	204 (14.4)	399 (26.7)	<0.001
	Occasional physical activity or sport (%)	386 (24.8)	452 (32.0)	258 (17.3)	
	Physical activity several times a month (%)	578 (37.1)	271 (19.2)	400 (26.8)	
	Sports or physical training several times a week (%)	407 (26.1)	485 (34.3)	436 (29.2)	
PA guidelines	Non-meeting (%)	1151 (73.9)	927 (65.7)	1057 (70.8)	<0.001
	Meeting (%)	407 (26.1)	485 (34.3)	436 (29.2)	
ST duration (min)	Mean (SD)	94.3 (94.3)	94.3 (77.1)	120.0 (102.9)	<0.001
ST guidelines	Non-meeting (%)	850 (54.6)	780 (55.2)	863 (57.8)	0.167
	Meeting (%)	708 (45.4)	632 (44.8)	630 (42.2)	
Sleep duration (hour)	Mean (SD)	9.0 (1.0)	9.0 (1.0)	9.0 (1.0)	0.343
Sleep guidelines	Non-meeting (%)	506 (32.5)	488 (34.6)	523 (35.0)	0.285
	Meeting (%)	1052 (67.5)	924 (65.4)	970 (65.0)	
Poverty	Mean (SD)	12.7 (2.6)	20.9 (3.8)	31.1 (2.7)	<0.001

BMI, body mass index; HEI, Healthy Eating Index; PA, physical activity; SD, standard deviation; SES, socioeconomic status; ST, screen time.

**Table 3:** Generalized linear mixed model examining the association of socioeconomic status and poverty status with overweight/obesity in Spanish children and adolescents.

		Outcome (Overweight/Obesity)	
Predictors		OR (Univariable)	OR (Multilevel)
*Specific individual average effects*			
SES status	Low SES	Reference	Reference
	Medium SES	0.62 (0.53–0.71, *p* < 0.001)	0.63 (0.54–0.73, *p* < 0.001)
	High SES	0.58 (0.49–0.68, *p* < 0.001)	0.59 (0.50–0.71, *p* < 0.001)
Immigrant status	Native-born	Reference	Reference
	Foreign-born	1.26 (0.94–1.66, *p* = 0.116)	1.12 (0.83–1.49, *p* = 0.462)
Age	Mean (SD)	0.96 (0.95–0.98, *p* < 0.001)	0.96 (0.94–0.98, *p* < 0.001)
Sex	Boys	Reference	Reference
	Girls	1.00 (0.88–1.13, *p* = 0.975)	1.01 (0.88–1.15, *p* = 0.938)
HEI status	Low HEI	Reference	Reference
	Medium HEI	0.88 (0.75–1.02, *p* = 0.092)	0.93 (0.79–1.09, *p* = 0.369)
	High HEI	0.88 (0.76–1.03, *p* = 0.122)	0.89 (0.75–1.05, *p* = 0.161)
PA category	No exercise	Reference	Reference
	Occasional physical activity or sport	0.96 (0.79–1.16, *p* = 0.658)	1.13 (0.92–1.39, *p* = 0.250)
	Physical activity several times a month	0.82 (0.68–0.99, *p* = 0.044)	1.06 (0.86–1.31, *p* = 0.586)
	Sports or physical training several times a week	0.80 (0.66–0.96, *p* = 0.019)	1.04 (0.85–1.28, *p* = 0.721)
ST guidelines	No	Reference	Reference
	Yes	0.97 (0.85–1.10, *p* = 0.588)	0.98 (0.86–1.12, *p* = 0.803)
Sleep guidelines	No	Reference	Reference
	Yes	0.82 (0.72–0.94, *p* = 0.004)	0.91 (0.79–1.05, *p* = 0.201)
*Specific contextual average effects*			
Poverty status	Low poverty	Reference	Reference
	Medium poverty	1.08 (0.92–1.27, *p* = 0.347)	1.10 (0.87–1.39, *p* = 0.410)
	High poverty	1.48 (1.27–1.73, *p* < 0.001)	1.48 (1.17–1.86, *p* = 0.001)
POOR (%)	Low poverty vs. Medium poverty		30.3
POOR (%)	Low poverty vs. High poverty		2.1
Country variance			0.018 (0.014–0.022)
ICC (%)			0.53
MOR			1.14 (1.12–1.15)
*Model performance metrics*			
BIC			5524.4
Nakagawa’s *R*^2^			0.041

BIC, Bayesian information criterion; HEI, Healthy Eating Index; ICC, intraclass correlation coefficient; MOR, median odds ratio; OR, odds ratio; PA, physical activity; POOR, Proportion of Opposite Odds Ratios; SD, standard deviation; SES, socioeconomic status; ST, screen time.

**Table 4:** Generalized linear mixed model examining the association of socioeconomic status and poverty status with obesity in Spanish children and adolescents.

		Outcome (Obesity)	
Predictors		OR (Univariable)	OR (Multilevel)
*Specific individual average effects*			
SES status	Low SES	Reference	Reference
	Medium SES	0.57 (0.46–0.70, *p* < 0.001)	0.56 (0.45–0.70, *p* < 0.001)
	High SES	0.39 (0.30–0.52, *p* < 0.001)	0.37 (0.28–0.50, *p* < 0.001)
Immigrant status	Native-born	Reference	Reference
	Foreign-born	1.58 (1.07–2.26, *p* = 0.016)	1.29 (0.88–1.91, *p* = 0.194)
Age	Mean (SD)	0.85 (0.83–0.87, *p* < 0.001)	0.85 (0.82–0.87, *p* < 0.001)
Sex	Boys	Reference	Reference
	Girls	0.95 (0.79–1.14, *p* = 0.588)	0.99 (0.82–1.20, *p* = 0.931)
HEI status	Low HEI	Reference	Reference
	Medium HEI	0.78 (0.62–0.98, *p* = 0.033)	0.76 (0.60–0.96, *p* = 0.022)
	High HEI	0.96 (0.77–1.20, *p* = 0.747)	0.81 (0.64–1.03, *p* = 0.091)
PA category	No exercise	Reference	Reference
	Occasional physical activity or sport	0.75 (0.58–0.97, *p* = 0.028)	0.92 (0.70–1.22, *p* = 0.564)
	Physical activity several times a month	0.50 (0.38–0.65, *p* < 0.001)	0.85 (0.63–1.15, *p* = 0.293)
	Sports or physical training several times a week	0.49 (0.38–0.64, *p* < 0.001)	0.95 (0.71–1.28, *p* = 0.749)
ST guidelines	No	Reference	Reference
	Yes	0.93 (0.77–1.13, *p* = 0.477)	0.93 (0.76–1.13, *p* = 0.443)
Sleep guidelines	No	Reference	Reference
	Yes	0.57 (0.47–0.69, *p* < 0.001)	0.86 (0.70–1.06, *p* = 0.168)
*Specific contextual average effects*			
Poverty status	Low poverty	Reference	Reference
	Medium poverty	1.02 (0.80–1.30, *p* = 0.876)	0.98 (0.71–1.35, *p* = 0.891)
	High poverty	1.48 (1.18–1.84, *p* = 0.001)	1.40 (1.02–1.94, *p* = 0.039)
POOR (%)	Low poverty vs. Medium poverty		45.9
POOR (%)	Low poverty vs. High poverty		6.0
Country variance			0.024 (0.019–0.029)
ICC (%)			0.61
MOR			1.16 (1.14–1.18)
*Model performance metrics*			
BIC			3026.4
Nakagawa’s *R*^2^			0.164

BIC, Bayesian information criterion; HEI, Healthy Eating Index; ICC, intraclass correlation coefficient; MOR, median odds ratio; OR, odds ratio; PA, physical activity; POOR, Proportion of Opposite Odds Ratios; SD, standard deviation; SES, socioeconomic status; ST, screen time.

## Data Availability

The data for this study were provided by the Ministry of Health, Consumer Affairs, and Social Welfare and are publicly accessible on the official Spanish Government website.
